# Based on Network Pharmacology and Molecular Docking to Explore the Underlying Mechanism of Huangqi Gegen Decoction for Treating Diabetic Nephropathy

**DOI:** 10.1155/2021/9928282

**Published:** 2021-05-06

**Authors:** Shanshan Ding, Weihao Wang, Xujiao Song, Hao Ma

**Affiliations:** ^1^School of Chemical and Biological Engineering, Yichun University, Yichun, Jiangxi 336000, China; ^2^School of Aesthetic Medicine, Yichun University, Yichun, Jiangxi 336000, China

## Abstract

**Background:**

Huangqi Gegen decoction (HGD), a Chinese herb formula, has been widely used to treat diabetic nephropathy in China, while the pharmacological mechanisms are still unclear. Therefore, the present study aims to explore the underlying mechanism of HGD for treating diabetic nephropathy (DN).

**Materials and Methods:**

Traditional Chinese Medicine Systems Pharmacology Database (TCMSP), UniProt, and SwissTargetPrediction databases were used to search the active ingredients and potential targets of HGD. In addition, multiple disease-related databases were used to collect DN-related targets. Common targets of the protein-protein interaction (PPI) network were established using the STRING database. Gene Ontology (GO) and Kyoto Encyclopedia of Genes and Genomes (KEGG) pathway enrichment analyses were performed using the DAVID database. At last, AutoDockVina was used to conduct molecular docking verification for the core components and targets.

**Results:**

A total of 27 active ingredients and 354 putative identified target genes were screened from HGD, of which 99 overlapped with the targets of DN and were considered potential therapeutic targets. Further analysis showed that the HGD activity of quercetin, formononetin, kaempferol, isorhamnetin, and beta-sitosterol ingredients is possible through VEGFA, IL6, TNF, AKT1, and TP53 targets involved in TNF, toll-like receptors, and MAPK-related pathways, which have anti-inflammatory, antiapoptosis, antioxidation, and autophagy effects, relieve renal fibrosis and renal cortex injury, and improve renal function, thus delaying the development of DN. The molecular docking results showed that quercetin, formononetin, kaempferol, isorhamnetin, beta-sitosterol had a good binding activity with VEGFA, IL6, TNF, AKT1, and TP53.

**Conclusion:**

This study demonstrated that HGD might take part in the treatment of DN through multicomponent, multitarget, and multichannel combined action.

## 1. Introduction

Diabetic nephropathy (DN) is one of the most important microvascular complications of diabetes, accounting for about 20%–40% of all diabetic patients [1]. It has become the main cause of chronic kidney disease and end-stage renal disease. The main clinical manifestations were persistent proteinuria, hyperglycemia, hypertension, edema, and decreased glomerular filtration rate. The etiology and pathogenesis of DN are still unclear. It is believed that DN is caused by multiple factors such as long-term hyperglycemia, glomerular ultrafiltration, extracellular matrix deposition, activation of the polyol pathway, accumulation of advanced glycation end products, protein kinase C activation, oxidative stress, and genetic factors [2]. At present, the clinical treatment for DN is limited, and it is still mainly to control blood sugar, reduce blood pressure, and reduce urinary protein.

Huangqi Gegen decoction (HGD), derived from ancient books, has the effects of invigorating the spleen and replenishing Qi, nourishing Yin and clearing heat, invigorating body fluid, and relieving thirst. It can be used in hypertension and diabetes due to deficiency of both Qi and Yin. The whole prescription is composed of *Astragalus membranaceus* (Huangqi) and *Pueraria* (Gegen). It has been found that *Astragalus membranaceus* has a therapeutic effect on DN characterized by renal insufficiency and pathological changes by reducing blood glucose and antioxidation [3]. *Pueraria* extract exerts an antidiabetic effect by activating a variety of mechanisms, such as reducing insulin resistance, increasing insulin release, inhibiting glucose absorption and reabsorption, and improving insulin sensitivity, glucose uptake, and metabolism [4]. HGD can reduce fasting blood glucose and improve insulin resistance in diabetic rats [5]. However, the specific mechanism and related pathways of the action of HGD related to DN remain elusive.

**Figure 1:**
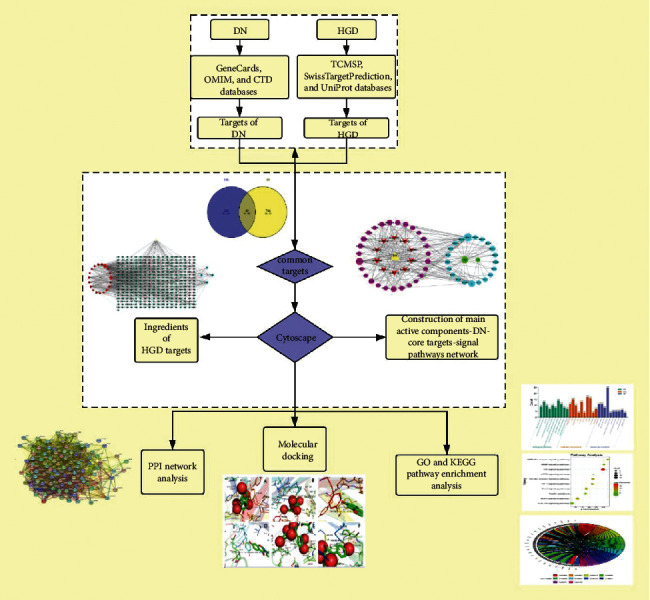
Flow diagram of the research.

Network pharmacology integrates multidisciplinary technology and content to construct a drug-target-disease network to explore the relationship between diseases and drugs [6]. At the same time, molecular docking promotes the pharmacological action to the molecular level and reveals the mechanism of drug action at the molecular level. Network pharmacology combined with molecular docking can make up for one-sided and unsystematic research on the mechanism of drug therapy. Therefore, this study explored the mechanism of HGD in the treatment of DN through network pharmacology method and molecular docking technology and provided new ideas for clinical application. Our flowchart is shown in Figure 1.

## 2. Materials and Methods

### 2.1. Database and Software

(Table 1)

### 2.2. Collection of Active Ingredients and Potential Targets

The active components of *Astragalus membranaceus* and *Pueraria* were obtained by searching “Huangqi” and “Gegen” in the TCMSP database. According to absorption, distribution, metabolism, and excretion (ADME) parameters, the active components of *Astragalus membranaceus* and *Pueraria* were obtained under the conditions of oral bioavailability (OB) ≥ 30% and drug-likeness (DL) ≥ 0.18, combined with related literature reports. The potential target information of active components was retrieved one by one through the UniProt database to obtain the corresponding gene names of drug targets. For other active ingredients that have not been retrieved, first, the 2D structure of their chemical components was downloaded from the PubChem website and saved in SDF format and then submitted to the SwissTargetPrediction database one by one to obtain the corresponding target of compounds.

### 2.3. Construction of Active Components-Target Network

The active components and drug targets of HGD obtained in 2.2 were used to construct the active ingredients-target network through Cytoscape software.

### 2.4. Collection of DN Disease Targets

Here, “diabetic nephropathy” was used as the keyword to search DN-related targets in three databases: GeneCards, OMIM, and CTD. In the integration of disease targets, the larger the score value of the target is, the closer the relationship between the target and the disease is. Therefore, the target is screened with score ≥10 as the standard in the GeneCards database, and the target database of DN is established by combining the targets in the three databases.

### 2.5. Core Target Screening and PPI Construction

Using the online tool of Venny, the potential targets of active components and the disease targets of DN were introduced, and the predictive targets of active components of HGD acting on DN were screened, and Venn maps were drawn. Upload the prediction target to the STRING database, limit the species to “*Homosapiens*”, select the medium confidence data with score condition greater than 0.40, hide the isolated proteins in the map, and finally derive the visual PPI network map. At the same time, the network is imported into Cytoscape to further analyze the core targets.

### 2.6. GO and KEGG Pathway Enrichment Analysis

DAVID database was used to analyze the enrichment of GO and KEGG pathways, and the gene type was “official gene symbol,” and the species type was “*Homo sapiens*.” Finally, GO functional annotation and KEGG pathway enrichment analyses were performed using the Weshengxin online platform, and a *P* value less than 0.05 was employed for further analysis.

### 2.7. Construction of Main Active Components-DN-Core Targets-Signal Pathways Network

The main active components, DN key targets, core targets, and signal pathways of HGD were introduced into Cytoscape to construct the network of main active components-DN key targets-core targets-signal pathways.

### 2.8. Molecular Docking

The core components of HGD were docked with the top five targets of the degree in 1.5. Firstly, the 3D structure of the target protein was downloaded from the PDB database, and the unique ligands similar to small molecules were found as protoligands. The target protein is then removed from water molecules and original ligands by PyMOL and introduced into AutoDockTools for hydrogenation; its charge is calculated; then, it is combined with nonpolar hydrogen and and saved into “pdbqt” format. Finally, AutoDock Vina was run for molecular docking, and PyMOL was used to visualize the results.

## 3. Results

### 3.1. Active Components and Potential Targets of HGD

Twenty-seven active components of *Astragalus membranaceus* and 18 active components of *Pueraria* were found by the TCMSP database. After screening by ADME parameters, 20 active components of *Astragalus membranaceus* and four active components of *Pueraria* were obtained. By consulting the literature, it was found that *Astragalus* polysaccharides can inhibit the TGF-*β*/Smad pathway and protect the kidney in DN model rats [17]. According to the literature, it was found that astragaloside IV can inhibit glomerular mesangial overproliferation and renal fibrosis through the TGF-*β*1/Smad/miR-192 pathway in rat glomerular mesangial cells and DN model rats, thus reducing renal injury [18]. Moreover, it was found that puerarin can inhibit the expression of NOX4 in podocytes, alleviate kidney injury in DN mice, and protect the kidney of DN mice by reducing oxidative stress and inhibiting inflammation [19, 20]. Therefore, astragalus polysaccharides, astragaloside IV, and puerarin found in the literature were also included in the active components of HGD, a total of 27 active components (Table 2). There were 1176 targets corresponding to each active component of HGD, and a total of 354 targets were obtained after screening and deleting repetitive values.

### 3.2. Active Ingredient-Target Network

**Figure 2:**
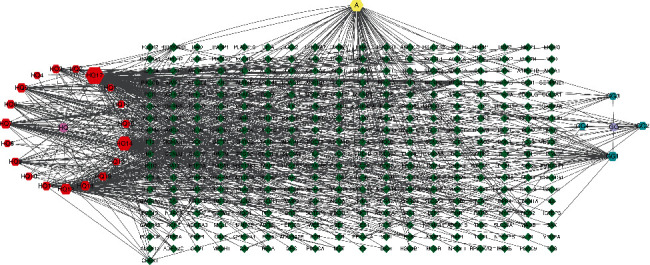
Active components-target network of potential targets of HGD. The hexagons represent the potential active components of HGD, and the diamonds represent the corresponding targets of the active components. Node area indicates its value: the larger the area, the more important the node.

Three hundred fifty-four targets and 27 active ingredients were imported into the Cytoscape software to construct the active ingredient-target network, and a network diagram composed of 351 nodes and 717 edges was obtained. The node size was proportional to its degree value, as shown in Figure 2.

### 3.3. Collection of DN Disease Targets

The DN target is obtained using three databases: GeneCards, OMIM, CTD. Among them, 3320 targets were obtained by GeneCards, and 655 targets were screened by GeneCards under the condition of score ≥10. Two hundred fifty-one targets were obtained by OMIM, and 114 targets were obtained by CTD. A total of 845 DN disease targets were obtained by combining and deduplication of the above targets.

### 3.4. Core Target Screening and PPI Construction

**Figure 3:**
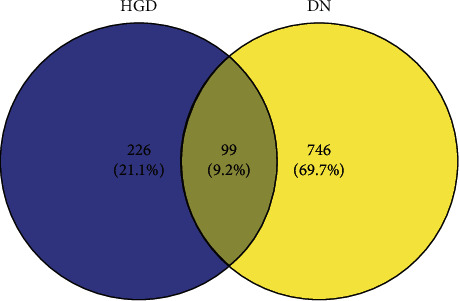
Active components of HGD-DN targets Venn diagram.

**Figure 4:**
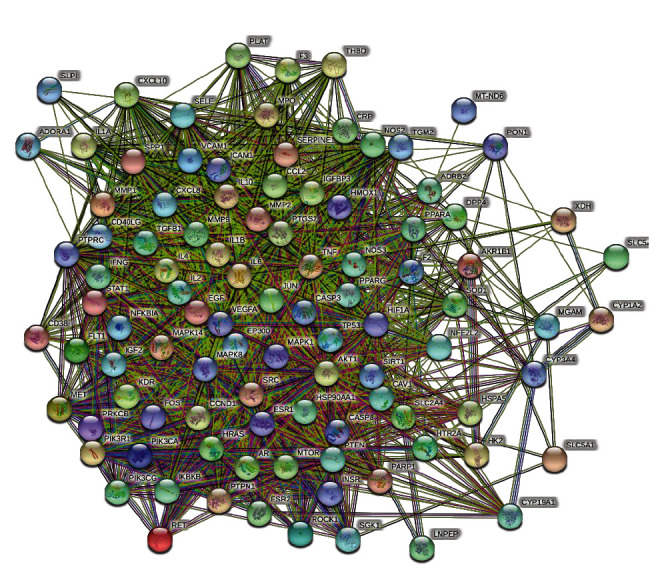
Common target PPI network between HGD and DN. Each bubble node represents a protein, and the 3D structure in the bubble nodes represents that the spatial protein structure is known or predicted. The lines among inner nodes display the relationship between different proteins, and the width of lines was based on the strength of data support.

The potential targets of active components of HGD were intersected with DN disease targets by Venny2.1.0 online tool, and finally, 99 main targets were obtained, and the Venn diagram was drawn, as shown in Figure 3. Upload the main targets to the STRING11.0 database to build the PPI network, as shown in Figure 4, which has 99 nodes and 1983 edges. The PPI results were imported into Cytoscape software and analyzed by the network analyzer. The results showed that the average degree of PPI was 40.06061, the average closeness centrality was 0.630402, and the average betweenness centrality was 0.006467. The targets that were all higher than the average were selected as the core targets of the active components of HGD acting on DN. A total of 27 targets were selected, as shown in Table 3. Among them, the top five target proteins were VEGFA, IL6, TNF, AKT1, and TP53, which indicated that the active components of HGD might treat DN through these targets.

### 3.5. GO and KEGG Pathway Enrichment Analysis

The above 27 core targets were uploaded to the DAVID database for enrichment analysis of GO and KEGG pathways. Setting *P* < 0.01, a total of 125 biological processes, 10 molecular functions, 13 cell components, and 81 signal pathways were obtained.

**Figure 5:**
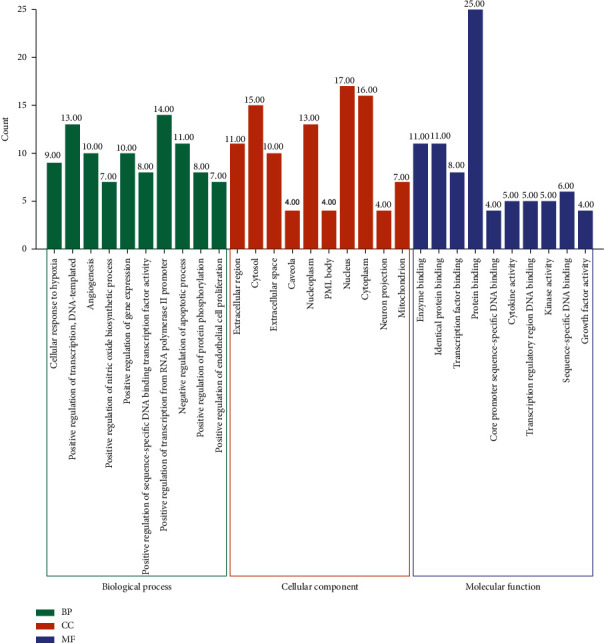
GO function analysis of active components of HGD acting on core targets of DN.

According to the size of the *P* value, the top 10 functional information sets are selected for analysis and mapping (Figure 5). The main biological processes involved in the core targets are a cellular response to hypoxia, positive regulation of transcription, angiogenesis, positive regulation of nitric oxide biosynthetic process, positive regulation of gene expression, positive regulation of sequence-specific DNA-binding transcription factor activity, positive regulation of transcription from RNA polymerase II promoter, negative regulation of the apoptotic process, positive regulation of protein phosphorylation, and positive regulation of endothelial cell proliferation. These results suggest that HGD may play an important role in the treatment of DN by regulating these biological processes. In addition, the main molecular functions involved are extracellular region, cytosol, extracellular space, caveola, nucleoplasm, PML body, nucleus, cytoplasm, neuron projection, and mitochondrion. At the same time, the main cellular components involved include enzyme binding, identical protein binding, transcription factor binding, protein binding, core promoter sequence-specific DNA binding, cytokine activity, transcription regulatory region DNA binding, kinase activity, sequence-specific DNA binding, and growth factor activity. These may play an important role in the occurrence and development of DN.

**Figure 6:**
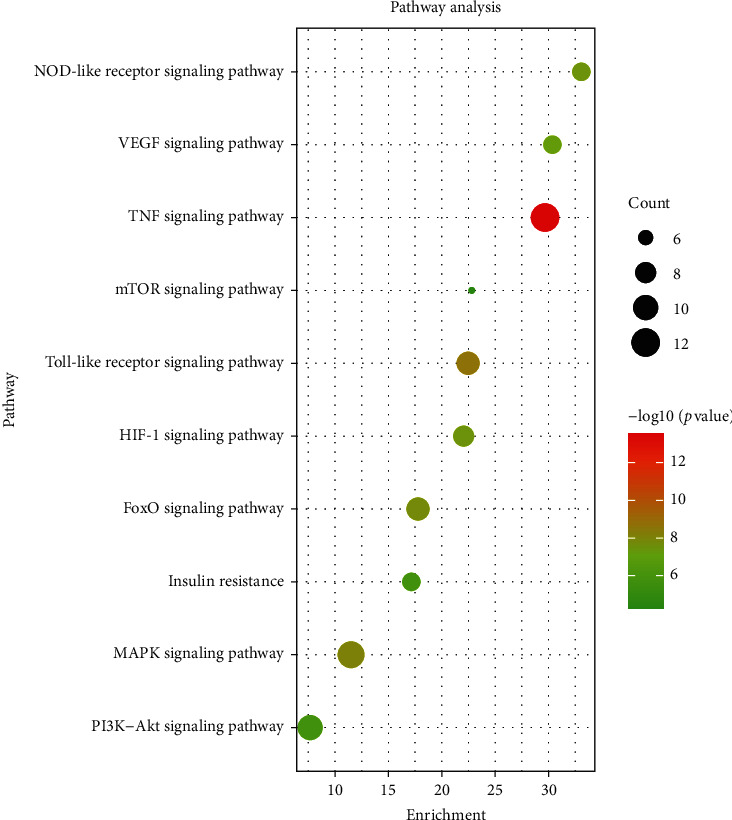
Bubble diagram of Kyoto Encyclopedia of Genes and Genomes analysis of active components of HGD acting on core targets of DN.

**Figure 7:**
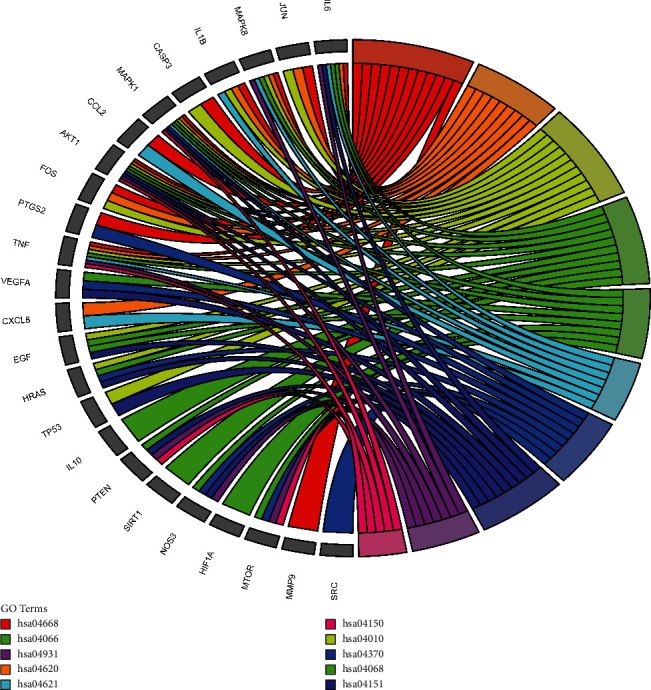
Kyoto Encyclopedia of Genes and Genomes annotation of active components of HGD acting on core targets of DN.

According to the size of the *P* value and the literature, the enrichment results of 10 KEGG pathways were selected. The specific information is shown in Table 4. It includes TNF signaling pathway, toll-like receptor signaling pathway, MAPK signaling pathway, FoxO signaling pathway, HIF-1 signaling pathway, NOD-like receptor signaling pathway, VEGF signaling pathway, PI3K-Akt signaling pathway, insulin resistance, and mTOR signaling pathway. The results showed that HGD alleviated the DN by regulating inflammatory reaction, autophagy, immunization, and antioxidant stress. Visualize the results, as shown in Figures 6 and 7 .

### 3.6. Construction of Main Active Components-DN-Core Targets-Signal Pathways Network

**Figure 8:**
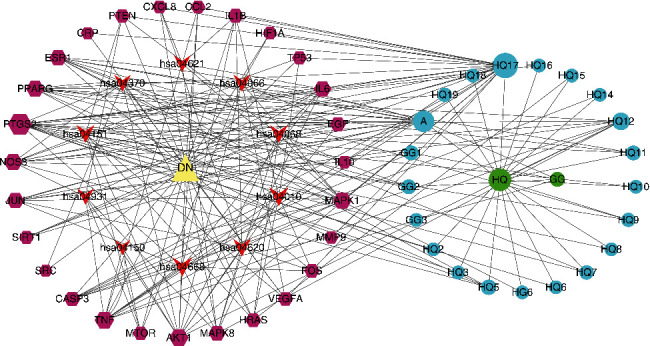
Active components of HGD-DN-core targets-signal pathways network. Circles represent HGD active components, hexagons represent targets, arrows represent signal pathways, and the triangle represents DN. Node area indicates its value: the larger the area, the more important the node.

The network of the active components-DN-core targets-signal pathways was constructed by Cytoscape software, as shown in Figure 8. The analysis results show that there are 61 nodes and 208 edges in the network. The larger the nodes, the more significant the impact of the node on DN. The quercetin degree, closeness centrality, and betweenness centrality are 23, 0.57142857, and 0.19158668, respectively. It is predicted that quercetin is an important component in the intervention of DN, followed by formononetin, kaempferol, isorhamnetin, and beta-sitosterol. Detailed information is shown in Table 5.

### 3.7. Molecular Docking Result

**Figure 9:**
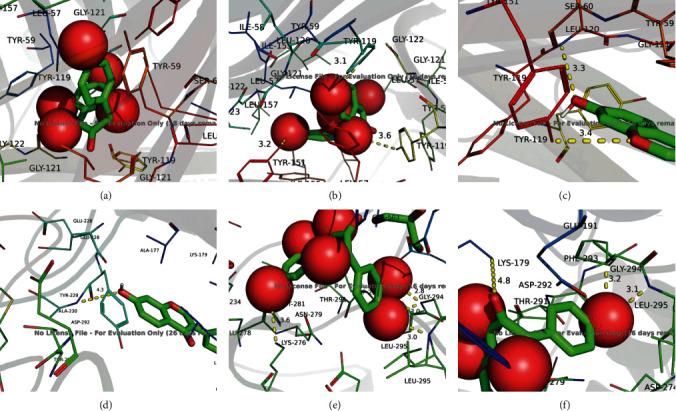
Molecular docking model. (a) Kaempferol-TNF, (b) quercetin-TNF, (c) formononetin-TNF, (d) formononetin-AKT1, (e) quercetin-AKT1, and (f) kaempferol-AKT1.

The core components in HGD were docking with the top five targets with a degree value of 1.5. The results showed that the binding energies were all less than −5 kcal/mol, indicating that they had good binding activity [21]. The specific information is shown in Table 6. Furthermore, the structure matching analysis is carried out using PyMoL software, as shown in Figure 9. The results showed that kaempferol formed *π*-*π* conjugation with TYR-59 amino acid residues in TNF, quercetin formed hydrogen bonds with TYR-151, TYR-119, and TYR-119 amino acid residues in TNF, formononetin formed hydrogen bonds with TYR-119 and LEU-120 amino acid residues in TNF, and formononetin formed hydrogen bonds with TYR-229 amino acid residues in AKT1. Quercetin forms a hydrogen bond with three amino acid residues of LYS-276, LEU-295, and GLY-294 in AKT1, and kaempferol forms a hydrogen bond with three amino acid residues of LYS-179, LEU-295, and GLY-294 in AKT1. These interactions make the structure tend to be stable.

## 4. Discussion

In this study, we screened out 27 active components of HGD and 99 targets related to DN by the network pharmacology method. It shows that HGD can play a role in treating DN through multicomponent and multitarget combination, which has research significance for DN treatment. According to the topological analysis results of the component-disease-core target-signal pathway network, the top five core components were quercetin, formononetin, kaempferol, isorhamnetin, beta-sitosterol.

Quercetin is a kind of flavonoid widely existing in various plants. It has anti-inflammatory and antioxidation activities, reduces blood glucose, blood pressure, and lipid, and has other pharmacological effects [22, 23]. It has been reported that quercetin can inhibit glomerular mesangial cell proliferation in DN mice by activating the Hippo pathway in DN mice, thereby reducing renal injury [24]. As a nonsteroidal polyphenol phytochemical, formononetin has a wide range of biological characteristics, involving anti-inflammatory, hypoglycemic, and other effects [25, 26]. Studies have shown that formononetin can improve glucose and lipid metabolism, reduce oxidative stress, and protect the kidney in diabetic db/db mice. Moreover, the inhibition of Smad3 protein and related regulatory factors of extracellular matrix could contribute to alleviation of renal fibrosis [27]. Kaempferol has anti-inflammation, antioxidation, antidiabetes, and antiaging effects [28]. In rat mesangial cells induced by advanced glycation end products, kaempferol could increase superoxide dismutase activity and decrease the content of malondialdehyde. It can inhibit the production of reactive oxygen species, the expression of type IV collagen and TGF-*β*1, and mitochondrial/cytochrome c-mediated apoptosis pathway and improve the recovery of mitochondrial membrane potential [29]. Isorhamnetin protects cardiocerebral vessels, has anti-inflammation and antioxidation effects, and prevents obesity [30]. A recent study showed that isorhamnetin protects the kidney by inhibiting the NF-*κ*B signaling pathway in diabetic rats [31]. Beta-Sitosterol has good antidiabetes and antioxidant effects in diabetic rats [32]. To sum up, most of these core components have anti-inflammatory, antioxidant, and hypoglycemic effects and can protect the kidney. The treatment of DN may be related to anti-inflammatory, antioxidant, and hypoglycemic effects.

In order to predict the mechanism of HGD in the treatment of DN, we carried out the enrichment analysis of the GO and KEGG pathways. The results showed that HGD could regulate the cellular response to hypoxia, positive regulation of transcription, angiogenesis, positive regulation of nitric oxide biosynthetic process, positive regulation of gene expression, and other biological processes, including extracellular region, cytosol, extracellular space, caveola, and nucleoplasm. At the same time, the activity is produced through molecular functions such as enzyme binding, identical protein binding, transcription factor binding, protein binding, and core promoter sequence-specific DNA binding. TNF signal pathway, toll-like receptor signal pathway, MAPK signal pathway, and other signal pathways are important pathways of HGD on DN, which are mainly involved in inflammation, immunity, autophagy, and oxidative stress.

The activation of the TNF signaling pathway can lead to glomerular vasoconstriction, a decrease of renal blood flow and glomerular filtration rate, and participation in the development of DN [33]. Moreover, TNF-*α* can cause inflammation of renal tissue, collagen deposition, and glomerular sclerosis, leading to kidney injury [34]. Toll-like receptor signaling pathway can promote the release of inflammatory factors such as IL-1, TNF-*α*, and MAPK, aggravate the inflammatory response, and lead to renal injury. It has been proved that toll-like receptor .0 induces the activation of NF-*κ*B, leading to renal cortex inflammation and renal fibrosis [35]. MAPK signaling pathway is related to inflammation, oxidative stress, cell differentiation, cell proliferation, and apoptosis. There are three major MAPK kinases in mammalian cells, namely, extracellular signal-regulated kinases (ERKs), c-Jun-N-terminal kinase, and p38MAPK. It was also found that ERKs and p38MAPK were activated in glomerular mesangial cells induced by high glucose and in glomeruli of DN mice, while activation of ERKs in high glucose environment led to renal mesangial cell hypertrophy [2, 36]. In summary, the above pathways are related to inflammation. In the pathogenesis of DN, inflammatory cells proliferate and differentiate, increase the infiltration of renal tissue cells, and secrete a large number of cytokines to mediate renal injury. Therefore, HGD in the treatment of DN is related to a series of inflammatory pathways.

HIF-1 signaling pathway mediates hypoxia-induced cell response by regulating genes involved in cell metabolism, glucose utilization, angiogenesis, oxidative stress, apoptosis, and proliferation, inhibits the expression of HIF-1 in a high glucose environment, affects podocyte survival, and leads to glomerular injury [37]. VEGF signaling pathway can stimulate endothelial cell proliferation and play a key role in physiological and pathological angiogenesis in different tissues. In the kidney, VEGF regulates glomerular permeability and maintains renal integrity, while DN is usually associated with impaired angiogenesis and capillary loss [38]. Studies have shown that the level of VEGF in the kidney of DN rats is increased. Inhibition of VEGF can alleviate the problems of glomerular hyperfiltration rate, glomerular hypertrophy, and urinary albumin excretion [39]. A series of studies have shown that activating PI3 K/Akt can not only inhibit the expression of mTOR protein, increase autophagy of renal cells, and improve renal function, but also increase the expression of FoxO protein and reduce the damage of renal podocytes induced by high glucose [40, 41]. In addition, molecular docking results showed that the core components of HGD had a strong binding activity with the AKT1 target. Therefore, these results also indicate that HGD can play a therapeutic role in DN by regulating oxidative stress and autophagy and counteracting hypoxia and other pathways.

## 5. Conclusions

In conclusion, the results of this study show that HGD can initially explore the potential mechanism of HGD in the treatment of DN through multitarget and multipathway combined action of DN. Through network pharmacology analysis, our study revealed that HGD might take part in the treatment of DN through pathways associated with insulin resistance, PI3K-Akt, toll-like receptors, MAPK, and TNF. Then, we validated the core targets and key components by molecular docking technology. The results showed that quercetin, formononetin, kaempferol, isorhamnetin, and beta-sitosterol had good binding activity with VEGFA, IL6, TNF, AKT1, and TP53. VEGFA, IL6, TNF, AKT1, and TP53 may be potential targets of HGD in treating DN. These results suggest that the antidiabetic nephropathy effect of HGD may be related to its direct or indirect regulation of the above potential targets and pathways, which provides a theoretical basis and research ideas for the clinical application of HGD in the treatment of DN. After all, so far, due to the complexity of traditional Chinese medicine compounds, although HGD has been used in the prevention and treatment of DN, its pharmacodynamic material basis and mechanism of action are still unclear. This study reveals the pharmacodynamic material basis and potential mechanism of HGD in the treatment of DN. Of course, some limitations exist in this study due to experimental conditions, time, and other constraints; we are unable to perform in vivo or in vitro experiments to validate the results in a short time. We will definitely conduct in-depth experimental research on the key targets and pathways of this article when conditions permit at a later period.

## Figures and Tables

**Table 1:** Involved databases and related analysis platforms in the study.

Name	Website	Version
TCMSP [7]	https://tcmspw.com/tcmsp.php	2.3
UniProt [8]	https://www.uniprot.org/	2020.8.3
PubChem [9]	https://pubchem.ncbi.nlm.nih.gov/	2019.3
SwissTargetPrediction [10]	http://www.swisstargetprediction.ch/	2020
GeneCards [11]	https://www.genecards.org/	5.0
OMIM [12]	https://www.omim.org/	2020.10.16
CTD [13]	http://ctdbase.org/	2020.10.5
STRING [14]	https://string-db.org/	11.0
DAVID [15]	https://David.ncifcrf.gov/	6.8
RCSB PDB [16]	http://www.rcsb.org	2020.11.24
Cytoscape	https://cytoscape.org/	3.7.2
Venny	https://bioinfogp.cnb.csic.es/tools/venny/	2.1.0
ChemDraw	http://www.adeptscience.de/	20.0
Chem3D	http://www.adeptscience.de/	20.0
PyMOL	https://pymol.org/2/	2.4.1
AutoDock Vina	http://vina.scripps.edu/	1.1.2
AutoDockTools	https://targetshttp://autodock.scripps.edu/	1.5.6

Abbreviations. TCMSP: Traditional Chinese Medicine Systems Pharmacology Database, OMIM: Online Mendelian Inheritance in Man, CTD: Comparative Toxicogenomics Database, DAVID: Database for Annotation, Visualization, and Integrated Discovery.

**Table 2:** Basic information of HGD active ingredients.

Sign	Mol ID	Molecule name	OB%	DL	Source
A	MOL000392	Formononetin	69.67	0.21	Huangqi and Gegen
GG1	MOL000358	Beta-Sitosterol	36.91	0.75	Gegen
GG2	MOL002959	3′-Methoxydaidzein	48.57	0.24	Gegen
GG3	MOL003629	Daidzein-4,7-diglucoside	47.27	0.67	Gegen
GG4	MOL012297	Puerarin	24.03	0.69	Gegen
HQ1	MOL000211	Mairin	55.38	0.78	Huangqi
HQ2	MOL000239	Jaranol	50.83	0.29	Huangqi
HQ3	MOL000296	Hederagenin	36.91	0.75	Huangqi
HQ4	MOL000033	(3 S,8 S,9 S,10 R,13R,14 S,17R)-10,13-Dimethyl-17-[(2R,5 S)-5-propan-2-yloctan-2-yl]-2,3,4,7,8,9,11,12,14,15,16,17-dodecahydro-1h-cyclopenta[a]phenanthren-3-ol	36.23	0.78	Huangqi
HQ5	MOL000354	Isorhamnetin	49.6	0.31	Huangqi
HQ6	MOL000371	3,9-di-O-Methylnissolin	53.74	0.48	Huangqi
HQ7	MOL000378	7-O-Methylisomucronulatol	74.69	0.3	Huangqi
HQ8	MOL000379	9,10-Dimethoxypterocarpan-3-O-*β*-D-glucoside	36.74	0.92	Huangqi
HQ9	MOL000380	(6aR,11aR)-9,10-Dimethoxy-6a,11a-dihydro-6h-benzofurano[3,2-c]chromen-3-ol	64.26	0.42	Huangqi
HQ10	MOL000387	Bifendate	31.1	0.67	Huangqi
HQ11	MOL000417	Calycosin	47.75	0.24	Huangqi
HQ12	MOL000422	Kaempferol	41.88	0.24	Huangqi
HQ13	MOL000433	FA	68.96	0.71	Huangqi
HQ14	MOL000438	(3R)-3-(2-Hydroxy-3,4-dimethoxyphenyl) chroman-7-ol	67.67	0.26	Huangqi
HQ15	MOL000439	Isomucronulatol-7,2′-di-O-glucosiole	49.28	0.62	Huangqi
HQ16	MOL000442	1,7-Dihydroxy-3,9-dimethoxy pterocarpene	39.05	0.48	Huangqi
HQ17	MOL000098	Quercetin	46.43	0.28	Huangqi
HQ18	-	Astragalus polysaccharide	-	-	Huangqi
HQ19	MOL000407	Astragaloside IV	22.50	0.15	Huangqi

Notes: the predicted targets of MOL000374 and MOL000398 cannot be corrected, so they are not included in the statistical data. Abbreviations. OB: Oral bioavailability. DL: Drug-likeness.

**Table 3:** Information about core targets and topology attributes.

Target name	Protein name	Degree	Closeness centrality	Betweenness centrality
VEGFA	Vascular endothelial growth factor A	84	0.875	0.03102384
IL6	Interleukin-6	84	0.875	0.04051797
TNF	Tumor necrosis factor	82	0.85964912	0.03718661
AKT1	RAC-alpha serine/threonine-protein kinase	82	0.85964912	0.04888032
TP53	Cellular tumor antigen p53	77	0.82352941	0.02116743
CASP3	Caspase-3	73	0.79674797	0.01770724
SRC	Proto-oncogene tyrosine-protein kinase Src	71	0.784	0.01916334
EGF	Pro-epidermal growth factor	70	0.765625	0.0160755
PTGS2	Alcohol dehydrogenase 1 B	69	0.765625	0.01023162
MAPK1	Mitogen-activated protein kinase 1	69	0.77165354	0.01315146
JUN	Transcription factor AP-1	67	0.75968992	0.00962486
CXCL8	Interleukin-8	66	0.7480916	0.01010843
MAPK8	Mitogen-activated protein kinase 8	66	0.75384615	0.00948131
MMP9	Matrix metalloproteinase-9	66	0.75384615	0.00961004
IL10	Interleukin-10	63	0.73684211	0.00935327
IL1 B	Interleukin-1 beta	62	0.72592593	0.01073204
CCL2	C-X-C motif chemokine 2	61	0.72592593	0.00853585
NOS3	Nitric-oxide synthase, endothelial	60	0.72058824	0.01623335
PTEN	Phosphatidylinositol-3,4,5-trisphosphate 3-phosphatase and dual-specificity protein phosphatase PTEN	60	0.72058824	0.0101365
MTOR	Serine/threonine-protein kinase mTOR	57	0.70503597	0.00941808
PPARG	Peroxisome proliferator-activated receptor gamma	56	0.7	0.00919915
ESR1	Estrogen receptor	56	0.7	0.01962422
FOS	Proto-oncogene c-Fos	54	0.69014085	0.01005477
SIRT1	Signal transducer and activator of transcription 1-alpha/beta	54	0.69014085	0.01725096
HRAS	GTPase HRas	54	0.69014085	0.00737461
CRP	C-reactive protein	49	0.66216216	0.00821785
HIF1A	Hypoxia-inducible factor 1-alpha	48	0.65771812	0.02349361

**Table 4:** Enrichment results of target pathway.

Id	KEGG pathway	*P* value	Targets	Count
Hsa04668	TNF signaling pathway	2.84*E* − 14	IL6, Jun, MAPK8, IL1 B, CASP3, MAPK1, CCL2, AKT1, FOS, PTGS2, TNF, MMP9	12
Hsa04620	Toll-like receptor signaling pathway	2.12*E* − 09	IL6, Jun, MAPK8, CXCL8, IL1 B, MAPK1, AKT1, FOS, TNF	9
Hsa04010	MAPK signaling pathway	7.62*E* − 09	Jun, MAPK8, EGF, IL1B, CASP3, MAPK1, AKT1, FOS, HRAS, TNF, TP53	11
Hsa04068	FoxO signaling pathway	1.38*E* − 08	IL10, IL6, MAPK8, EGF, PTEN, MAPK1, AKT1, HRAS, SIRT1	9
Hsa04066	HIF-1 signaling pathway	3.24*E* − 08	IL6, EGF, NOS3, MAPK1, AKT1, HIF1A, MTOR, VEGFA	8
Hsa04621	NOD-like receptor signaling pathway	3.48*E* − 08	IL6, MAPK8, CXCL8, IL1B, MAPK1, CCL2, TNF	7
Hsa04370	VEGF signaling pathway	5.88*E* − 08	Src, NOS3, MAPK1, AKT1, PTGS2, HRAS, VEGFA	7
Hsa04151	PI3K-Akt signaling pathway	1.81*E* − 06	IL6, EGF, NOS3, PTEN, MAPK1, AKT1, HRAS, TP53, MTOR, VEGFA	10
Hsa04931	Insulin resistance	1.81*E* − 06	IL6, MAPK8, NOS3, PTEN, AKT1, TNF, MTOR	7
Hsa04150	mTOR signaling pathway	5.04*E* − 05	Pten, MAPK1, AKT1, TNF, MTOR	5

**Table 5:** Specific information about the core components of HGD.

Sign	MOL ID	Molecule name	Degree	Closeness centrality	Betweenness centrality
HQ17	MOL000098	Quercetin	23	0.57142857	0.19158668
A	MOL000392	Formononetin	16	0.4379562	0.03963011
H12	MOL000422	Kaempferol	9	0.45801527	0.03824223
H5	MOL000354	Isorhamnetin	5	0.39735099	0.00766088
GG1	MOL000358	Beta-Sitosterol	4	0.38216561	0.01012611

**Table 6:** Results of molecular docking.

Core component	Target	PDB ID	Binding energy (kcal/mol)
	VEGFA	4QAF	−5.8
	IL6	1ALU	−5.4
Quercetin	TNF	6OP0	−10
	AKT1	4GV1	−7.9
	TP53	5O1E	−8.2
	VEGFA	4QAF	−9.4
	IL6	1ALU	−5.5
Formononetin	TNF	6OP0	−9.2
	AKT1	4GV1	−8.4
	TP53	5O1E	−7.6
	VEGFA	4QAF	−6.1
	IL6	1ALU	−5.7
Kaempferol	TNF	6OP0	−10.1
	AKT1	4GV1	−7.8
	TP53	5O1E	−7.0
	VEGFA	4QAF	−8.1
	IL6	1ALU	−5.2
Isorhamnetin	TNF	6OP0	−8.2
	AKT1	4GV1	−8.2
	TP53	5O1E	−8.0
	VEGFA	4QAF	−7.7
	IL6	1ALU	−5.1
Beta-Sitosterol	TNF	6OP0	−9.7
	AKT1	4GV1	−9.3
	TP53	5O1E	−6.1

## Data Availability

The data used in the study are available upon request to the corresponding author.

## References

[1] Alicic R. Z., Rooney M. T., Tuttle K. R. (2017). Diabetic kidney disease. *Clinical Journal of the American Society of Nephrology*.

[2] Bhattacharjee N., Barma S., Konwar N., Dewanjee S., Manna P. (2016). Mechanistic insight of diabetic nephropathy and its pharmacotherapeutic targets: an update. *European Journal of Pharmacology*.

[3] Han H., Cao A., Wang L. (2017). Huangqi decoction ameliorates streptozotocin-induced rat diabetic nephropathy through antioxidant and regulation of the TGF-*β*/MAPK/PPAR-*γ* signaling. *Cellular Physiology and Biochemistry*.

[4] Yang L., Chen J., Lu H. (2019). Pueraria lobatafor diabetes mellitus: past, present and future. *The American Journal of Chinese Medicine*.

[5] Chen Y. F., Wang C. Y., Li W. M. (2012). Effect of Huangqi gegen decoction (HGD) on TGF-beta1/Smad3 pathway in diabetic cardiomyopathy rats. *Zhong Yao Cai*.

[6] Kibble M., Saarinen N., Tang J., Wennerberg K., Mäkelä S., Aittokallio T. (2015). Network pharmacology applications to map the unexplored target space and therapeutic potential of natural products. *Natural Product Reports*.

[7] Ru J., Li P., Wang J. (2014). TCMSP: a database of systems pharmacology for drug discovery from herbal medicines. *Journal of Cheminformatics*.

[8] Consortium U. P. (2019). UniProt: a worldwide hub of protein knowledge. *Nucleic Acids Research*.

[9] Kim S., Chen J., Cheng T. (2019). PubChem 2019 update: improved access to chemical data. *Nucleic Acids Research*.

[10] Daina A., Michielin O., Zoete V. (2019). SwissTargetPrediction: updated data and new features for efficient prediction of protein targets of small molecules. *Nucleic Acids Research*.

[11] Stelzer G., Rosen N., Plaschkes I. (2016). The GeneCards suite:from gene data mining to disease genome sequence analyses. *Curr Protoc Bioinformatics*.

[12] Amberger J. S., Hamosh A. (2017). Searching online mendelian inheritance in man (OMIM):A knowledgebase of human genes and genetic phenotypes. *Nucleic Acids Research in Current Protocols Bioinformatics*.

[13] Davis A. P., Grondin C. J., Johnson R. J. (2019). The comparative toxicogenomics database: update 2019. *Nucleic Acids Research*.

[14] Szklarczyk D., Franceschini A., Kuhn M. (2011). The STRING database in 2011: functional interaction networks of proteins, globally integrated and scored. *Nucleic Acids Research*.

[15] Huang D. W., Sherman B. T., Lempicki R. A. (2009). Systematic and integrative analysis of large gene lists using DAVID Bioinformatics Resources. *Nature Protocols*.

[16] Burley S. K., Berman H. M., Kleywegt G. J., Markley J. L., Nakamura H., Velankar S. (2017). Protein data bank (PDB): the single global macromolecular structure archive. *Methods in Molecular Biology*.

[17] Meng X., Wei M., Wang D. (2020). Astragalus polysaccharides protect renal function and affect the TGF-*β*/Smad signaling pathway in streptozotocin-induced diabetic rats. *Journal of International Medical Research*.

[18] Mao Q., Chen C., Liang H., Zhong S., Cheng X., Li L. (2019). Astragaloside IV inhibits excessive mesangial cell proliferation and renal fibrosis caused by diabetic nephropathy via modulation of the TGF‑*β*1/Smad/miR‑192 signaling pathway. *Experimental and Therapeutic Medicine*.

[19] Li X., Cai W., Lee K. (2017). Puerarin attenuates diabetic kidney injury through the suppression of NOX4 expression in podocytes. *Scientific Reports*.

[20] Xu X., Zheng N., Chen Z., Huang W., Liang T., Kuang H. (2016). Puerarin, isolated from Pueraria lobata (Willd.), protects against diabetic nephropathy by attenuating oxidative stress. *Gene*.

[21] Li B., Rui J., Ding X., Yang X. (2019). Exploring the multicomponent synergy mechanism of Banxia Xiexin Decoction on irritable bowel syndrome by a systems pharmacology strategy. *Journal of Ethnopharmacology*.

[22] Li Y., Yao J., Han C. (2016). Quercetin, inflammation and immunity. *Nutrients*.

[23] Chen S., Jiang H., Wu X., Fang J. (2016). Therapeutic effects of quercetin on inflammation, obesity, and type 2 diabetes. *Mediators of Inflammation*.

[24] Lei D., Chengcheng L., Xuan Q. (2019). Quercetin inhibited mesangial cell proliferation of early diabetic nephropathy through the Hippo pathway. *Pharmacological Research*.

[25] Tay K.-C., Tan L. T.-H., Chan C. K. (2019). Formononetin: a review of its anticancer potentials and mechanisms. *Frontiers in Pharmacology*.

[26] El-Bakoush A., Olajide O. A. (2018). Formononetin inhibits neuroinflammation and increases estrogen receptor beta (ER*β*) protein expression in BV2 microglia. *International Immunopharmacology*.

[27] Lv J., Zhuang K., Jiang X., Huang H., Quan S. (2020). Renoprotective effect of formononetin by suppressing Smad3 expression in Db/Db mice. *Diabetes, Metabolic Syndrome and Obesity: Targets and Therapy*.

[28] Imran M., Rauf A., Shah Z. A. (2019). Chemo-preventive and therapeutic effect of the dietary flavonoid kaempferol: a comprehensive review. *Phytotherapy Research*.

[29] Jiang W., Wang R., Liu D. (2018). Protective effects of kaempferitrin on advanced glycation end products induce mesangial cell apoptosis and oxidative stress. *International Journal of Molecular Sciences*.

[30] Gong G., Guan Y.-Y., Zhang Z.-L. (2020). Isorhamnetin: a review of pharmacological effects. *Biomedicine & Pharmacotherapy*.

[31] Qiu S., Sun G., Zhang Y., Li X., Wang R. (2016). Involvement of the NF-*κ*B signaling pathway in the renoprotective effects of isorhamnetin in a type 2 diabetic rat model. *Biomedical Reports*.

[32] Gupta R., Sharma A. K., Dobhal M. P., Sharma M. C., Gupta R. S. (2011). Antidiabetic and antioxidant potential of *β*-sitosterol in streptozotocin-induced experimental hyperglycemia. *Journal of Diabetes*.

[33] Lampropoulou I. T., Stangou Μ., Sarafidis P. (2020). TNF-*α* pathway and T-cell immunity are activated early during the development of diabetic nephropathy in Type II Diabetes Mellitus. *Clinical Immunology*.

[34] Malik S., Suchal K., Khan S. I. (2017). Apigenin ameliorates streptozotocin-induced diabetic nephropathy in rats via MAPK-NF-*κ*B-TNF-*α* and TGF-*β*1-MAPK-fibronectin pathways. *American Journal of Physiology-Renal Physiology*.

[35] Han W., Ma Q., Liu Y. (2019). Huangkui capsule alleviates renal tubular epithelial-mesenchymal transition in diabetic nephropathy via inhibiting NLRP3 inflammasome activation and TLR4/NF-*κ*B signaling. *Phytomedicine*.

[36] Shao X., Kong W. X., Li Y. T. (2019). MiR-133 inhibits kidney injury in rats with diabetic nephropathy via MAPK/ERK pathway. *European Review for Medical and Pharmacological Sciences*.

[37] Bohuslavova R., Cerychova R., Nepomucka K., Pavlinkova G. (2017). Renal injury is accelerated by global hypoxia-inducible factor 1 alpha deficiency in a mouse model of STZ-induced diabetes. *BMC Endocrine Disorders*.

[38] Lin S., Teng J., Li J., Sun F., Yuan D., Chang J. (2016). Association of chemerin and vascular endothelial growth factor (VEGF) with diabetic nephropathy. *Medical Science Monitor*.

[39] Pang X., Zhang Y., Peng Z., Shi X., Han J., Xing Y. (2020). Hirudin reduces nephropathy microangiopathy in STZ-induced diabetes rats by inhibiting endothelial cell migration and angiogenesis. *Life Sciences*.

[40] Liu Q., Wang J. J., Hu Y. Q. (2020). Berberine regulates PI3K/AKT/FOXO1/Bim signaling pathway to improve high glucose-induced podocyte injury. *Zhong GuoYao Li Xue*.

[41] Qiu B. N., Xiong Y. A., Pan Y. F. (2020). The mechanism of oxymatrine alleviating oxidative damage of colonic mucosal cells by regulating autophagy in UC mice. *Tian Ran Yao Wu Yu Kai Fa*.

